# Evaluation of the Predominant Dietary Pattern and Sleep Disorders in Obese Diabetic Patients and Non-diabetic Obese Individuals: Short title: The Dietary Pattern and Sleep Disorders in Diabetics

**DOI:** 10.31661/gmj.v15i.3888

**Published:** 2026-06-29

**Authors:** Fatemeh Amiri, Homeira Rashidi, Alireza Sedaghat, Fatemeh Esmaeili

**Affiliations:** ^1^ Diabetes Research Center, Health Research Institute, Ahvaz Jundishapur University of Medical Sciences, Ahvaz, Iran

**Keywords:** Dietary Pattern, Diabetes Mellitus, Sleep Quality, Obesity

## Abstract

**Background:**

Objective: Diabetes mellitus is a chronic metabolic disorder and a major public health concern worldwide, particularly in developing countries. Dietary habits and sleep quality are important factors influencing metabolic health and diabetes outcomes. This study aimed to compare dietary patterns and sleep disorders among obese patients with diabetes and obese individuals without diabetes.

**Materials and Methods:**

This quasi-experimental study was conducted in 2023 on 140 obese adults, including 70 patients with diabetes and 70 non-diabetic individuals, who attended the endocrinology subspecialty clinic of Imam Khomeini Hospital in Ahvaz, Iran. Dietary intake was assessed using a 147-item Food Frequency Questionnaire (FFQ), while sleep quality and daytime sleepiness were evaluated using the Pittsburgh Sleep Quality Index (PSQI) and the Epworth Sleepiness Scale (ESS), respectively. Healthy, traditional, and Western dietary patterns were identified and compared between the groups.

**Results:**

No significant difference was observed in mean age between the two groups. However, diabetic participants had significantly higher body weight than non-diabetic subjects (P=0.02). Sleep quality was poor in both groups, but diabetic patients had significantly worse PSQI scores than non-diabetic individuals (13.35±1.26 vs. 9.41±0.98, P=0.001). Consumption of fruits, vegetables, and low-fat dairy products did not differ significantly between groups, whereas intake of high-fat dairy products was significantly higher among diabetic patients (P=0.03). Adherence to the traditional and Western dietary patterns was associated with a 4.42-fold and 3.63-fold increased risk of type 2 diabetes, respectively, while no significant association was found for the healthy dietary pattern.

**Conclusion:**

Obese patients with diabetes exhibit poorer sleep quality and less favorable dietary habits than obese non-diabetic individuals. These findings highlight the importance of promoting healthy dietary patterns and improving nutritional awareness to support diabetes prevention and management.

## Introduction

Diabetes mellitus is one of the major public health problems in the world, and its prevalence is increasing every year [1]. Diabetes mellitus is characterized by a disorder in the metabolism of carbohydrates, lipids, and proteins and is caused by low insulin secretion, insulin resistance [2].

Nutritional interventions are a key component of managing type 2 diabetes [3]. Making health-promoting changes in eating patterns can improve postprandial blood glucose cycles and reduce HbA1c to reduce diabetes-related complications and mortality [4].

According to research, a decrease in dietary diversity, an increase in refined foods and saturated fats, along with a lack of physical activity, are among the main factors in the increase in the prevalence and complications of diabetes [5]. Also, one of the most important methods of treating and preventing type 2 diabetes is weight loss, which is achieved by reducing daily calorie intake and increasing physical activity [6, 7]. Many cases of obesity and type 2 diabetes can be treated with the above two methods [5].

Short sleep and poor-quality sleep are associated with poor diet and physical activity levels. Since the mid-2000s, there has been increasing evidence linking short sleep duration (SSD) to obesity [8, 9]. Longitudinal studies have also shown that SSD is associated with greater weight gain than normal sleep [10]. Few studies in Iran have examined the relationship between high blood sugar and sleep quality [11]. In addition to metabolic outcomes, recent studies suggest that dietary patterns may also affect sleep quality. Poor sleep is both a consequence and potential contributor to impaired glucose metabolism. Diets high in saturated fats, sugar, and refined carbohydrates have been linked to shorter sleep duration, lower sleep quality, and increased risk of sleep disorders. Conversely, nutrient-dense diets may support better sleep quality, which in turn may help improve metabolic function in obese individuals [12].

Findings of a study showed that sleep duration was positively associated with body fat reduction after adjusting for age, sex, baseline BMI, intervention length, and change in energy intake, while sleep quality was inversely associated with body fat reduction, such that those with poor sleep quality had less body fat reduction [13].

By reviewing previous studies and considering the very rapid increase in the number of patients with type 2 diabetes mellitus, especially in developing countries, as well as the importance of optimal blood sugar control in diabetics by adjusting lifestyle, the importance of paying attention to sleep hygiene and psychological status in people with diabetes is raised as a strategy for optimal prevention and treatment of diabetic patient [14].

The present study aimed to compare sleep disorders, and food frequency in obese patients with diabetes with obese individuals without diabetes.

## Materials and Methods

This quasi-experimental study was conducted on 70 obese patients with type 2 diabetes and 70 obese non-diabetic individuals referred to the endocrinology subspecialty clinic of Imam Khomeini Hospital in Ahvaz, Iran, in 2023.

Inclusion criteria were: Age between 18 and 70 years, Body mass index (BMI) ≥30 kg/m² (defined as obese), Diagnosis of type 2 diabetes (for the diabetic group) confirmed by a specialist based on ADA criteria, Absence of diabetes in the non-diabetic group confirmed through medical history and self-reported data.

Exclusion criteria included: History of respiratory diseases (e.g., rhinitis, asthma, bronchitis), Severe cardiovascular diseases (e.g., acute myocardial infarction, heart failure), Neurological or psychiatric disorders (e.g., major depressive disorder, epilepsy, schizophrenia), Diagnosed sleep disorders (e.g., obstructive sleep apnea), Use of medications known to affect sleep or appetite (e.g., sedatives, corticosteroids), Any severe physical or mental illness that could interfere with participation, Pregnancy or lactation, Lack of informed consent. This exclusion framework was applied to reduce the potential influence of confounding factors on both sleep quality and dietary behavior. Participants were selected using purposive sampling from eligible outpatients.

In order to collect data, a demographic questionnaire (age, gender, marital status, occupation, and financial status), a 147-item food frequency questionnaire (FFQ), and Pittsburgh Sleep Quality Questionnaire were used.

147-item FFQ: Individuals' intake of various types of snacks was collected and evaluated using a semi-quantitative food frequency questionnaire, so that intake of nuts and dried fruits was considered as healthy snacks and intake of salty and sweet snacks was considered as unhealthy snack consumption. Specifically, we used factor analysis on the FFQ to identify three major dietary patterns: Healthy dietary pattern, characterized by high consumption of vegetables, fruits, whole grains, legumes, and low-fat dairy products. Traditional dietary pattern, which included high intake of refined grains, rice, tea, red meat, and saturated fats. Western dietary pattern, marked by frequent consumption of processed foods, sugary beverages, high-fat dairy products, fast foods, and sweets.

The 8-question questionnaire ESS measures the likelihood of a person dozing off during various daily activities. The range of scores is between 0 and 24. A total score of 11 or more indicates severe sleepiness and a high risk of obstructive sleep apnea.

Pittsburgh Sleep Quality Index (PSQI) Questionnaire: This questionnaire assesses individuals' perceptions of sleep quality over the past four weeks. It has seven items. Each item on the questionnaire is scored from zero to three. The components include: 1. The individual's overall description of sleep quality 2. Delay in falling asleep 3. Duration of useful sleep 4. Sleep adequacy (calculated based on the ratio of useful sleep duration to total time spent in bed) 5. Sleep disturbances (measured as the individual's nighttime awakenings) 6. Amount of sleeping medication consumed 7. Daily functioning (defined as problems caused by poor sleep experienced by the individual during the day). Seven components should be examined in PSQI scoring. The minimum and maximum scores considered for each component range from 0 (no problem) to 3 (very serious problem). Finally, the scores for each component were added together and converted into a total score (0 to 21). A high score in each component or in the total score indicates poor sleep quality. Scores of 0-1-2-3 in each scale indicate a normal state, mild, moderate, and severe problems, respectively. The sum of the scores on the seven scales forms the total score, which ranges from zero to 21. A total score of 6 or more means poor sleep quality.

The PSQI and ESS were selected due to their validated use in the Iranian population, ease of administration, and strong reliability and validity in assessing sleep quality and daytime sleepiness. These tools are also widely used in studies involving individuals with obesity and metabolic disorders, making them appropriate for the present research [15].

Statistical Analysis

Statistical analysis was performed by SPSS software Version 22 . Mean±SD and number (percentage) indicate quantitative and qualitative variables, respectively. Kolmogorov-Smirnov and Shapiro-Wilk tests were used for normality distribution. For statistical analysis, chi-square tests, independent t-test, one-way analysis of variance or its nonparametric equivalent (Kruskal-Wallis test) were used. To examine the relationship between the variables and effectiveness, the logistic regression model were used, respectively. P<0.05 was considered statistically significant.

## Results

**Table:** Table1. Demographic and Clinical Characteristics of Participants

Variable		Obese patients with diabetes (n=70)	Non-diabetic obese individuals (n=70)	P-value*
Age, year		41.76±9.04	29.70±11.60	0.24
Weight, Kg		100.60±14.81	94.54±16.84	0.02
Height, cm		172.00±10.62	164.27±22.86	0.01
BMI, kg/m2		33.90±2.31	33.34±2.45	0.55
Sex, n (%)	Male	37 (52.90)	26 (37.10)	0.062
	Female	33 (47.10)	44 (62.90)	
	Single	47 (67.10)	57 (81.40)	0.21
Marital status , n (%)	Married	22 (31.40)	13 (18.60)	
	Divorced	1 (1.40)	0	
Occupation, n (%)	self-employment	18 (25.70)	18 (25.70)	0.99
	Governmental	52 (74.30)	52 (74.30)	
Smoking, n (%)		47 (67.10)	57 (81.40)	0.053
Hypertension, n (%)		29 (41.40)	22 (31.40)	0.21
Dyslipidemia, n (%)		49 (70.0)	21 (30.0)	0.001

^*^ Independent t-test and chi-square were applied; significant P-values were bolding (P<0.05).

**Table:** Table2. Findings Related to Dietary Intake

Energy intake from macronutrients	Obese patients with diabetes (n=70)	Non-diabetic obese individuals (n=70)	P-value*
Daily energy intake (kcal)	3945.24±198.79	3180.65±221.21	0.01
Carbohydrates	648.70±234.56	541.81±286.74	0.017
Protein	159.97±61.69	127.54±67.69	0.004
0Fat	151.82±66.63	125.27±66.46	0.02
Whole grains	87.88±126.87	97.63±157.65	0.68
Refined grains	747.47±299.09	570.58±274.94	0.001
Red meat	34.56±29.16	25.10±20.55	0.02
White meat and eggs	100.11±63.09	91.68±59.89	0.41
Processed meats	13.93±25.96	6.95±8.69	0.03
Fast food	84.48±121.57	45.61±60.25	0.01
Vegetables	473.91±237.76	510.14±364.01	0.48
Fruits	472.90±334.91	420.29±449.91	0.79
Juice	89.07±113.26	78.91±211.23	0.72
Legumes	80.67±52.27	82.36±79.87	0.88
Pickles and salt	83.21±96.79	68.05±72.76	0.29
Nuts	33.99±35.87	41.33±60.70	0.38
Solid oils	18.74±18.66	17.07±18.27	0.59
Liquid oils	30.59±19.35	23.61±20.13	0.03
Sweets	125.63±122.99	120.64±123.61	0.81
Seasonings	17.71±10.53	10.37±8.29	0.001
Caffeinated foods	585.12±363.29	526.84±339.31	0.32
High-fat dairy products	286.75±232.61	206.59±213.26	0.03
Low-fat dairy products	145.59±115.16	172.55±158.26	0.25
Unhealthy snacks	19.51±29.09	17.30±26.58	0.63

^*^ Independent t-test was applied; significant P-values were bolding (P<0.05).

**Table:** Table3. Factor Loadings of Food Groups in the Identified Exploratory Dietary Patterns

Food groups	Healthy dietary pattern	Traditional dietary pattern	Western dietary pattern
Fruits	0.70		
Liquid oil	0.67		
Whole grains	0.66		
Vegetables	0.64		
Low-fat dairy products	0.63		
Caffeinated drinks	0.59		
Nuts and seeds	0.54		
Natural juice	0.30		
Beans	0.45		
White meat and eggs		0.73	
Pickles and salt		0.69	
Refined grains		0.66	
High-fat dairy products		0.60	
Processed meats			0.91
Red meat			0.86
Fast food			0.54
Sweets and sweet drinks			0.28
Solid oil			0.14
Unhealthy snacks			0.11
Percentage of variance explained	20.46%	11.99%	11.32%

**Table:** Table4. Association between Dietary Patterns and Odds of Diabetes

	Crude model		
Dietary Pattern			
Healthy Pattern	Odds ratio (95% CI)	Standard Error	P-value *
1st tertile (Low adherence)		Reference level	
2nd tertile (Moderate adherence)	2.72 (1.11 – 4.64)	0.45	0.27
3rd tertile (High adherence)	2.08 (0.96 – 4.49)	0.39	0.06
	Adjusted with weight height, dyslipidemia		
1st tertile (Low adherence)		Reference level	
2nd tertile (Moderate adherence)	0.85 (0.3 -2.38)	0.52	0.76
3rd tertile (High adherence)	0.43 (0.15 – 1.20)	0.51	0.11
	Crude model		
Traditional pattern			
1st tertile (Low adherence)		Reference level	
2nd tertile (Moderate adherence)	1.51 (0.65 – 3.49)	0.42	0.33
3rd tertile (High adherence)	4.42 (1.84 – 10.56)	0.44	0.001
	Adjusted with weight height, dyslipidemia		
1st tertile (Low adherence)		Reference level	
2nd tertile (Moderate adherence)	2.16 (0.81 – 5.75)	0.5	0.12
3rd tertile (High adherence)	5.33 (1.96 – 14.46)	0.5	0.001
	Crude model		
Western pattern			
1st tertile (Low adherence)		Reference level	
2nd tertile (Moderate adherence)	1.37 (0.6 – 3.16)	0.42	0.44
3rd tertile (High adherence)	3.63 (1.54-8.57)	0.43	0.003
	Adjusted with weight height, dyslipidemia		
1st tertile (Low adherence)		Reference level	
2nd tertile (Moderate adherence)	1.56 (0.58 – 4.15)	0.5	0.37
3rd tertile (High adherence)	5.54 ( 1.98 – 15.46)	0.52	0.001

^*^ Logistic regression was applied; significant P-values were bolding (P<0.05).

**Table:** Table5. Comparison of Sleepiness Levels between the Two Groups according to Demographic Characteristics

Variable	Non-diabetic obese individuals	Obese patients with diabetes	P-value *
Epworth Sleepiness Scale (ESS)	7.65 ± 5.13	9.01 ± 5.58	0.13
	Based on the sex		
Female	6.95 ± 4.51	9.96 ± 4.90	0.98
Male	8.88 ± 5.96	10.83 ± 5.58	0.19
	Based on the hypertension		
Yes	9.90 ± 5.64	10.62 ± 4.90	0.67
No	6.59 ± 4.58	7.87 ± 4.72	0.20
	Based on the dyslipidemia		
Yes	9.00 ± 5.62	8.77 ± 5.75	0.88
No	7.06 ± 4.84	9.57 ± 5.25	0.07
	Based on the smoking status		
Yes	9.92 ± 6.37	10.30 ± 6.34	0.86
No	7.12 ± 4.71	8.38 ± 5.12	0.19

^*^ chi-square test was applied; significant P-values were bolding (P<0.05).

**Table:** Table6. Pittsburgh’s Sleep Quality between Two Groups and according to Demographic Information

Variable	Non-diabetic obese individuals	Obese patients with diabetes	P-value *
Sleep quality (PSQI)	9.41±0.98	13.35±1.26	0.001
	Based on the sex		
Female	12.07±2.48	12.16±3.46	0.90
Male	12.08±3.24	13.80±3.01	0.50
	Based on the hypertension		
Yes	12.10±2.97	11.60±2.68	0.15
No	13.46±3.73	12.84±2.54	0.25
	Based on the dyslipidemia		
Yes	11.53±2.80	13.23±4.54	0.01
No	12.40±2.67	13.00±2.08	0.24
	Based on the smoking status		
Yes	12.66±3.25	14.39±2.51	0.02
No	11.77±3.40	11.80±2.54	0.11

^*^ Independent t-test and chi-square were applied; significant P-values were bolding (P<0.05).

A total of 140 participants were enrolled, equally divided between obese individuals with diabetes (n=70) and non-diabetic obese individuals (n=70). The table below provides a summary of the comparison of clinical and demographic factors. Although the difference was not statistically significant (P=0.24), the mean age of the diabetic group was greater (41.76±9.04 years) than that of the non-diabetic group (29.70±11.60 years). The diabetic group's weight (100.60±14.81 kg) was substantially greater than that of the non-diabetic group (94.54±16.84 kg), showing a statistically significant difference (P=0.02). Likewise, there was a statistically significant difference in height (P=0.01). However, there was no significant difference in BMI between the groups (P=0.55). In terms of the distribution of both sexes the diabetic group had a greater proportion of men (52.9%) than the non-diabetic group (37.1%), but this difference was not statistically significant (P=0.062). Marital status, occupation, smoking status, and hypertension did not differ statistically significantly. There is a strong correlation between dyslipidemia and diabetes in obese people, as evidenced by the substantial difference in the prevalence of dyslipidemia, which was larger in the diabetic group (70.0%) than in the non-diabetic group (30.0%), P=0.001.

The dietary intake study showed that the total daily energy consumption of obese people with diabetes was considerably higher than that of obese people without diabetes (3945.24±198.79 kcal vs. 3180.65±221.21 kcal, P=0.01). All of the main macronutrients showed this variation. The group with diabetes consumed considerably more carbohydrates (648.70±234.56 g) than the group without diabetes (541.81±286.74 g, P=0.017). Similarly, diabetics consumed higher amounts of fat (151.82±66.63 g vs. 125.27±66.46 g, P=0.02) and protein (159.97±61.69 g vs. 127.54±67.69 g, P=0.004). Regarding certain dietary groups, the diabetic group consumed considerably more refined grains (747.47±299.09 g) than the non-diabetic group (570.58±274.94 g, P=0.001). They also reported consuming more seasonings (17.71±10.53 g vs. 10.37±8.29 g, P=0.001), high-fat dairy products (286.75±232.61 g vs. 206.59±213.26 g, P=0.03), and liquid oils (30.59±19.35 g vs. 23.61±20.13 g, P=0.03). Consumption of whole grains, white meat and eggs, vegetables, fruits, juices, legumes, pickles and salt, nuts, solid oils, low-fat dairy products, sweets, caffeinated foods, and unhealthy snacks did not differ statistically significantly between the two groups (P>0.05 for all comparisons). These data demonstrate that obese diabetes individuals tend to have a higher overall calorie intake and consume greater amounts of macronutrients, notably refined carbs, proteins, and fats. These results are provided in Table2. The study population's three main food trends were determined using Principal Component Analysis (PCA). Table3 displays the retrieved patterns together with the factor loadings that correlate to them. Based on earlier research, the following names were given to the eating patterns and their constituent parts: High consumption of fruits, liquid oils, whole grains, vegetables, low-fat dairy products, caffeinated beverages, nuts and seeds, natural fruit juices, and seasonings were all features of the healthy eating pattern. Of the entire variation, 20.46% was explained by this pattern. White meat and eggs, pickles and salt, refined cereals, and high-fat dairy items were all part of the traditional diet. A total of 11.99% of the variance was explained by this pattern. High intakes of processed meats, red meat, fast food, sugar-sweetened beverages, organ meats, and solid fats were characteristics of the Western eating pattern. Of the entire variation, 11.32% was explained by this pattern. A detailed interpretation of the data regarding the association between dietary patterns and odds of diabetes, based on both the crude and adjusted models show in the Table4. In healthy dietary pattern, the risks of having diabetes were 2.72 times greater for those in the second tertile of adherence to a healthy dietary pattern in the crude model than for those in the first tertile (poor adherence); however, this difference was not statistically significant (P=0.27). In the analysis of the healthy dietary pattern, no significant association was found between adherence to this pattern and the risk of type 2 diabetes, both in the crude model and in models adjusted for potential confounders (weight, height, and dyslipidemia). One possible explanation for this lack of significance is that although participants reported some elements of healthy eating, the intensity or consistency of adherence to this pattern may have been insufficient to influence metabolic outcomes. Additionally, overlaps with other dietary patterns and lifestyle factors not captured in the FFQ may have diluted the effect. Additionally, the odds were higher for those in the third tertile (high adherence) (OR=2.08), with a marginally significant difference (P=0.06). The higher adherence groups had lower risk of diabetes after controlling for height, weight, and dyslipidemia. The adjusted odds ratio was 0.85 (P=0.76) in the second tertile and 0.43 (P=0.11) in the third. After controlling for confounding variables, this indicates a possible protective tendency of good dietary adherence, even though it is not statistically significant. In the traditional dietary pattern, moderate adherence was associated with non-significantly higher odds (OR=1.51, P=0.33) in the crude model. However, with an odds ratio of 4.42 (95% CI: 1.84–10.56, P=0.001), those in the third tertile (high adherence) had a noticeably increased chance of developing diabetes, suggesting a robust link between traditional dietary practices and the disease. This connection was still significant after adjustment. Regardless of height, weight, or lipid profile, the third tertile's odds ratio increased to 5.33 (95% CI: 1.96–14.46, P=0.001), indicating the strong association between high adherence to a traditional diet and a higher risk of diabetes. Moderate Western diet adherence did not significantly correlate with the crude model (OR=1.37, P=0.44). Nonetheless, there was a significant correlation between higher chances of diabetes and high adherence (third tertile) (OR=3.63, 95% CI: 1.54–8.57, P=0.003). This association strengthened further after adjustment, with the odds ratio increasing to 5.54 (95% CI: 1.98–15.46, P=0.001), showing a strong and statistically significant link between Western dietary patterns—characterized by high intakes of processed meats, red meats, fast food, and solid fats—and a higher risk of diabetes, even after controlling for anthropometric and metabolic confounders. Based on the results in the Table5, obese patients with diabetes had a higher mean ESS score than non-diabetic obese people (9.01 ± 5.58 vs. 7.65 ± 5.13). Men with diabetes also reported higher drowsiness levels than their non-diabetic counterparts (10.83 ± 5.58 vs. 8.88 ± 5.96), while women with diabetes had higher scores than non-diabetic women (9.96 ± 4.90 vs. 6.95 ± 4.51). These variations were not statistically significant, though (P=0.19 for men and P=0.98 for women). Participants with hypertension reported higher ESS ratings in both groups, according to their status. The mean score of hypertensive diabetics was 10.62 ± 4.90, while that of non-diabetics was 9.90 ± 5.64. However, the difference was not statistically significant (P=0.67). Diabetic participants reported higher levels of tiredness (7.87 ± 4.72 vs. 6.59 ± 4.58), although this difference was not statistically significant (P=0.20) compared to those without hypertension. The drowsiness scores of patients with dyslipidemia did not differ significantly from one another (P=0.88). Diabetics, however, reported higher ESS scores than non-diabetics (9.57 ± 5.25 vs. 7.06 ± 4.84), with a near-significant trend (P=0.07) among those without dyslipidemia. Individuals with diabetes and those without the disease who smoked reported feeling more sleepy than those who did not smoke. The average score for smokers with diabetes was 10.30 ± 6.34, while the score for non-diabetics was 9.92 ± 6.37 (P=0.86). The diabetic group's nonsmokers also reported feeling more sleepy than the non-diabetic group's nonsmokers (8.38 ± 5.12 vs. 7.12 ± 4.71, P=0.19).

Sleep quality in non-diabetic individuals and diabetic patients and was 9.41±0.98 and 13.35±1.26 respectively, and the results showed that both groups had poor sleep quality, but the sleep quality of diabetic patients was significantly worse(P=0.001). More details are provided in Table6.

## Discussion

The current study aimed to investigate and compare sleep disturbances and meal frequency in obese patients with diabetes with obese individuals without diabetes. Despite the importance of regulating dietary factors for effective diabetes management and the existence of guidelines on diabetic diets, there is still little information about the actual dietary patterns of diabetic individuals in Iran.

Diabetic patients in this study consumed significantly more macronutrients, carbohydrates, protein, and fat compared to non-diabetics. There was no significant difference in vegetable and fruit intake between diabetic patients and non-diabetics (P=0.48, P=0.79). High-fat dairy consumption was higher in diabetic patients than in non-diabetics (P=0.03), while low-fat dairy consumption was not significantly different between the two groups (P=0.25).

The poorer sleep quality observed among diabetic patients in this study may be explained by underlying metabolic and physiological mechanisms associated with type 2 diabetes. Previous research has demonstrated a bidirectional relationship between sleep disturbances and impaired glucose metabolism. Poor sleep quality has been linked to increased sympathetic nervous system activity, elevated evening cortisol levels, and decreased insulin sensitivity, all of which contribute to insulin resistance and suboptimal glycemic control [16]. Additionally, chronic sleep disruption may promote systemic inflammation and exacerbate metabolic dysfunction. These findings support the plausibility of the sleep impairments observed in our diabetic participants and highlight the importance of incorporating sleep health into diabetes management strategies.

A study by Saaty et al. investigated dietary patterns in patients with type 2 diabetes compared to non-diabetic controls and reported that diabetics had significantly higher intake of vegetables, fruits, whole grains, and healthy fats such as olive oil (P<0.001) [17]. These findings suggest that diabetic individuals may adopt healthier eating behaviors after diagnosis, possibly due to medical advice or increased awareness. In contrast, our study did not find a significant difference in fruit and vegetable consumption between diabetic and non-diabetic obese individuals, which may reflect lower adherence to dietary guidance or differences in cultural and regional dietary habits. This discrepancy highlights the importance of not only providing nutritional education but also reinforcing adherence and individualized support in dietary management, especially in clinical settings.

In a study by Ueno et al., three or more servings of vegetables per day were inversely associated with type 2 diabetes [18]. Fatima et al. also reported in their study that antioxidants such as polyphenols, carotenoids, and vitamin C, which are abundant in fruits and vegetables, are associated with a lower incidence of T2DM [19]. These findings suggest a potential protective role of plant-based nutrients in glycemic regulation and inflammation reduction. It also underscores the possibility that overall dietary patterns, rather than isolated food groups, might play a more critical role in diabetes development.

According to a meta-analysis by Wang et al., eating more raw fruits and vegetables, mainly green leafy vegetables, has the potential to reduce the risk of developing type 2 diabetes [20]. The results of these studies are inconsistent with the findings of the current study. This discrepancy may be due to differences in sample size, study design, and underlying diseases.

In some studies it was shown that there were a weak inverse association between vegetable consumption and type 2 diabetes [21, 22]. One of the reasons the lack of a statistically significant association between vegetable consumption and type 2 diabetes in our study may be due to the inherently weak association between these variables, which may not reach significance in the study populations.

Other studies have found that consumption of fruits, vegetables alone, or a combination of both did not significantly reduce the risk of T2DM [23]. However, in our study, no significant difference was observed in the consumption of fruits and vegetables between diabetic and non-diabetic obese individuals. This may be due to the relatively weak association between vegetable intake and diabetes risk, which may not reach statistical significance in smaller or more homogeneous samples.

Sanders et al. concluded in a meta-analysis that increased consumption of red and processed meat is associated with an increased risk of type 2 diabetes [24].

Zelber et al. also found that eating meat, especially red and processed meat, significantly increased the risk of insulin resistance and nonalcoholic fatty liver disease. Increased saturated fat intake was associated with insulin resistance, impaired fasting glucose, and higher glucose concentrations in a 2-hour oral glucose tolerance test [25]. These outcomes may be explained by the high saturated fat content in these meats, which contributes to metabolic dysfunction and impaired glucose regulation. In our study, individuals following Western and traditional dietary patterns—which typically include higher consumption of red and processed meats—had significantly higher odds of developing type 2 diabetes. These findings are consistent with the literature and reinforce the harmful metabolic effects of high saturated fat and animal protein consumption in obese populations.

Malin et al. found that eating whole grains was associated with significantly lower postprandial glucose and insulin levels compared with eating refined grains [26]. Whole grain consumption was associated with a lower risk of prediabetes [27].

Our findings showed that refined grain consumption was higher in diabetic patients, while whole grain intake was not significantly different between the two groups. This pattern may contribute to poorer glycemic control and increased insulin resistance, potentially exacerbating the progression of type 2 diabetes. The higher refined grain consumption observed in diabetic individuals might reflect habitual dietary behaviors established prior to diagnosis or a lack of adherence to dietary recommendations post-diagnosis. These findings reinforce the importance of promoting dietary patterns rich in whole grains and low in refined carbohydrates as part of diabetes prevention and management strategies. A meta-analysis study that examined the effects of soda and fruit juice consumption on type 2 diabetes found that soda and fruit juice consumption increased the risk of diabetes [28].

Another meta-analysis found that sugar-sweetened beverage consumption was associated with a fivefold increased risk of abdominal obesity in diabetics, which was associated with increased insulin resistance and increased risk of cardiovascular disease [29]. According to the current study, the consumption of caffeinated beverages did not show a significant difference between the two groups, which may indicate the similarity of consumption habits of these foods between non-diabetics and diabetics. This finding may suggest that caffeine-containing drinks—many of which are unsweetened, such as tea or coffee—may not be a primary contributor to dietary differences observed between the groups. Alternatively, it could reflect similar cultural or habitual consumption patterns across the population, regardless of diabetes status.

It is also possible that while overall caffeine consumption was similar, differences in the type and sugar content of beverages (e.g., sweetened vs. unsweetened coffee or tea) were not fully captured, which may limit the ability to detect dietary influences on metabolic health in this category. Further investigation into the specific types of caffeinated beverages consumed and their preparation methods may offer greater insight into their role in diabetes risk and management.

Diabetes, as a common metabolic disease, adversely affects aspects of life and sleep quality [30].

According to the findings of our study, the mean sleepiness score in diabetic patients (5.58 ± 9.01) was higher than that in non-diabetic obese subjects (5.65 ± 7.13), but this difference was not statistically significant (P=0.13). The overall comparison of sleep quality between diabetic patients and non-diabetic obese subjects showed that both groups had poor sleep quality, but the condition of diabetic patients was significantly worse (P=0.001).

Sadakhati’s study concluded that diabetics have more delayed nocturnal sleep than normal individuals due to impaired glucose levels [31]. Joukar et al. showed that diabetics have lower sleep quality compared to healthy individuals due to impaired sleep onset and duration of nocturnal sleep [32]. Another study showed a U-shaped relationship between sleep duration and hemoglobin A1c levels, with both short and long sleep durations being associated with increased hemoglobin A1c levels compared to normal sleep [33]. According to a study by Zeighami et al, individuals who had poor sleep quality or had difficulty falling asleep early had a higher relative risk of developing type 2 diabetes [34]. These findings imply that sleep disturbances may not only be a consequence of diabetes but also a contributing factor in its development and progression.

Taken together, our results support the growing body of evidence indicating that sleep quality should be considered an integral component of diabetes management. The significantly poorer sleep quality observed among diabetic patients in our study may be attributed to physiological mechanisms such as insulin resistance, inflammation, and autonomic dysfunction, all of which are known to be associated with both sleep disruption and metabolic disorders.

Short sleep duration at night increases sympathetic system activity, increases nocturnal cortisol levels, disrupts carbohydrate metabolism, and increases growth hormone levels during the day. The result of these changes is reduced glucose tolerance and increased peripheral tissue resistance to insulin, which increases the risk of developing diabetes [35]. Sleep disruption also reduces serum leptin and increases blood ghrelin, both of which lead to impaired glucose control and, at the same time, to worsening of the disease [36].

The results of these studies fully confirm the current results. In the current study, sleep quality between diabetic patients and non-diabetic obese individuals showed that both groups had poor sleep quality, but the condition of diabetic patients was significantly worse.

The summary of the findings of this study, in line with previous studies, indicates the importance of paying attention to sleep hygiene and the psychological state of these patients as a prevention and treatment strategy. Therefore, considering the poor quality of sleep of these patients, it is recommended to educate the patient on the necessary measures or eliminate the factors affecting sleep disorders.

One limitation of this study is the observed variation in weight and height between groups, despite all participants meeting the BMI criteria for obesity. Future studies should consider matching or adjusting for these variables to enhance comparability and reduce potential confounding. Although the FFQ used in this study has been validated in the general Iranian population, it has not been specifically validated in obese individuals with and without diabetes. This may affect the accuracy of dietary intake reporting in these subgroups and should be considered a limitation of the study.

## Conclusion

Overall, our findings showed that diabetic patients have a higher energy and fat diet, higher consumption of refined grains, fast foods, red and processed meat, and condiments, which can negatively affect their disease control. This consumption pattern highlights the need to modify eating habits and increase nutritional awareness in diabetic patients. Our results also indicated poor sleep quality in patients with diabetes. One of the limitations of the study is the small sample size and the single-center nature of the study. It is recommended that further multicenter studies with larger sample sizes be conducted to confirm these results. Healthcare providers can use these findings to promote healthier dietary patterns and screen for poor sleep quality in diabetic patients. Targeted education and lifestyle interventions can improve both glycemic control and overall well-being.

## Conflict of Interest

There is no conflict of interest.

## AI Disclosure Statement

During the preparation of this manuscript, the authors used ChatGPT, OpenAI company for language editing, grammar improvement, and liboberry.com for reference management. After its use, the authors thoroughly reviewed, verified, and revised all AI-assisted content to ensure accuracy and originality. The authors take full responsibility for the integrity and final content of the published article.
